# Can economic and environmental benefits associated with agricultural intensification be sustained at high population densities? A farm level empirical analysis

**DOI:** 10.1016/j.landusepol.2018.10.046

**Published:** 2019-02

**Authors:** Daniel Kyalo Willy, Milu Muyanga, Thomas Jayne

**Affiliations:** a Kenyatta University, Department of Agribusiness Management and Trade, PO Box 43844, Nairobi, Kenya; b Department of Agricultural, Food and Resource Economics, Michigan State University, East Lansing, MI 48824, USA

**Keywords:** Population density, Sustainable intensification, Soil quality, Crop productivity, Smallholder agriculture

## Abstract

*Boserup’s* pioneering theory holds that rising population density can be accompanied by sustainable agricultural intensification. But can this positive relationship be sustained indefinitely, or are there conditions under which rising population density can lead to declining agricultural productivity? This study utilizes survey data on farm households in Kenya and soil samples on their main maize plots to assess whether Boserupian agricultural intensification is sustainable at high population densities. The study employs econometric estimation methods to assess the effect of land management practices and population density on soil quality and then determines the effect of soil quality on crop productivity. Results show evidence of endogenous sustainable agricultural intensification accompanied by improvements in soil quality and crop yields at low population densities. However, as population densities exceed roughly 600 persons/km^2^, we observe a deterioration in indicators of soil organic and reactive carbon, soil pH, and plant available phosphorous. Deterioration in soil quality leads to binding nutrient constraints associated with reduced crop yield response to inorganic fertilizer application that further reduces crop productivity. These results raise the specter of unsustainable forms of agricultural intensification associated with deteriorating soil capital, and point to the imperative of identifying and implementing effective strategies for increasing farmers’ use of sustainable land management practices in rural areas facing already high and rising population densities.

## 1 Introduction

Population density growth is a critical agricultural development issue because of its impact on crop production through its effect on land availability and quality. There is a growing concern about the potential impacts of further population density growth especially in sub-Saharan Africa where the majority of people depend on rain-fed smallholder agriculture. Drawing from the Boserupian theory (Boserup, 1965), some studies have found evidence that mounting population densities induce agricultural intensification. For example, the widely acclaimed “Machakos Miracle” as presented by Tiffen et al. (1994) portrayed a success story where growing population density was accompanied by *Boserupian* agricultural intensification. Adoption of new seed and fertilizer technologies supported agricultural intensification and productivity growth, attracting more intensive use of labor and capital per unit of cultivated land (Mortimore et al., 1991; Mortimore and Tiffen, 1994). Economic dividends associated with the population density-induced agricultural intensification were reinvested in water and soil conservation interventions that reversed land degradation and further raised crop yields. The seminal work of Tiffen et al. (1994) provides evidence that sustainable intensification can accompany rising population density.

Later studies emerged to recast how farming systems intensify in the face of growing population density. When evaluating the ‘Machakos Miracle’, critics have claimed that the effects observed by Tiffen et al. (1994) could have been confounded by the effect of proximity of Machakos to Nairobi, a major urban center. Zaal and Oostendorp (2002) argued that the distance to urban centers was as important as population density in explaining the driving force to investments in land conservation strategies. Proximity to cities provides a ready market for agricultural output, the proceeds of which can be reinvested to support further agricultural intensification. Access to urban wage work can also be used to also facilitate further farm intensification. Therefore, the widespread intensification reported in Machakos was viewed by some as being a result of urban influence, institutional and policy factors, and not necessarily being driven by population density alone. Further, Murton (1997, 1999), questioned the use of highly aggregated data which is likely to mask differences in social and economic impacts associated with environmental changes. Nevertheless, all the studies seem to converge on the importance of achieving sustainable agricultural intensification.

Over 20 years have passed since the Machakos Miracle was documented. Machakos has since then undergone substantial transformations as population densities continue to rise. Some parts of the county report population densities of over 1000 persons/km^2^ (KNBS, 2010). Farming systems have also changed along with declining farm sizes and significant changes in land use and land cover. These changes are not unique to Machakos; they are characteristic of many increasingly densely populated areas of sub-Saharan Africa where agriculture remains a key livelihood source. Many regions in rural Kenya, especially the central and western highlands, now exceed the population densities recorded in Machakos during the 1990s. Further, it has been reported that although the green revolution had substantial success in saving forests and wetlands, and improving peoples livelihoods, the productivity gains associated with it have since slowed (Pingali, 2012). More recently, studies by Muyanga and Jayne (2014) and Ricker-Gilbert et al. (2014) have identified population density thresholds beyond which the productivity gains associated with agricultural intensification start to vanish. These are not very encouraging findings especially considering that most parts of sub-Saharan Africa are set to experience rising rural population densities until at least 2050.

Considering this situation, two major research questions emerge. First, given the rate of technical innovation experienced in the area, has the positive relationship between agricultural intensification and population density as reported in the Tiffen et al Machakos study from the 1980s been sustained at the much higher levels of population density that this county is now experiencing? Second, what could be the driving force behind potential population density thresholds beyond which agricultural productivity declines? In the quest to seek answers to these questions, this study evaluates the trends in agricultural intensification in the context of growing population densities; estimates the effect of population density, land management practices, plot attributes and institutional factors on soil quality; and assess how crop yields have responded to changes in soil quality in densely populated areas.

The contribution of the current study to the population growth-agricultural intensification literature is three-fold. First, we test the ‘more people less erosion’ hypothesis using plot-level data to control for plot heterogeneity. Second, we assess the influence of agricultural intensification on the quality of soil and crop yields while controlling for urban influence, institutional, and policy factors. To control for urban influence, Kisii, a county with favorable agro-ecological potential similar to Machakos but considerably more distant from a major urban center, is included in the study to identify the influence of urban proximity on intensification. Third, the current study combines socio-economic data and plot level soil quality data in assessing the link between population density and agricultural productivity. We seldom encounter such approaches in literature despite the importance of soil quality in explaining yield response to input use. The study therefore takes a rare approach that incorporates plot level soil sample data into socioeconomic analysis to evaluate the sustainability of agricultural intensification in the face of growing population density. We revisit the Tiffen et al. (1994) study sites to assess how the acclaimed population density-induced agricultural intensification has evolved over the 20-year period since this influential study was carried out.

## 2 Description of the study area and data

**Fig. 1:**
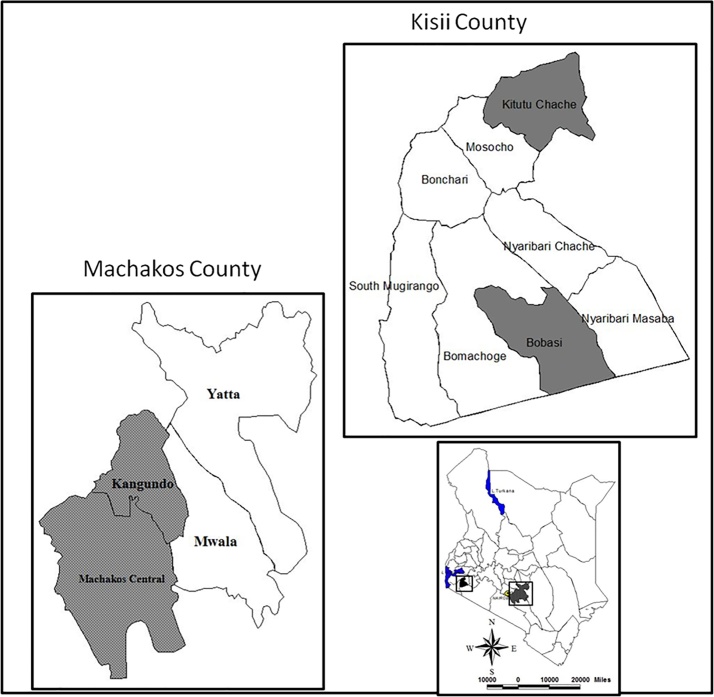
Map of Study areas.

The study was conducted in Machakos and Kisii Counties, Kenya (Fig. 1). Machakos County is located in Eastern Kenya at latitudes 0° 45′ South to 1° 31′ South and longitudes 36° 45′ East to 37° 45′ East, with an altitude range between 1000 and 1600 m above sea level (m.a.s.l). The county covers approximately 6208 km^2^, with an estimated population of 1,098,584 persons, according to the 2009 Kenya National Bureau of Statistics (KNBS) Kenya Population and Housing Census (Republic of Kenya, 2010). Kisii County is located South East of Lake Victoria in Western Kenya, at latitude 0° 41′ 0 S and longitude 34° 46′ 0 E. The county covers an estimated area of 1317 km^2^, with a population of 1,152,282 persons and an average population density of 874 people per km^2^ (Republic of Kenya, 2010).

The primary data used in this study were obtained through a cross-sectional survey among 290 randomly selected farm households located in densely populated regions of Machakos and Kisii Counties in February to April 2014. Within each County, sub-Counties were purposively selected based on population density estimates obtained from the KNBS population census data (Republic of Kenya, 2010). After the selection of the sub-Counties, a multistage random sampling procedure was followed to select households. First, a random sample of Wards^1^ was drawn, followed by a random sample of sub-locations and then villages. From each village, a sampling frame was developed with the help of village elders, from which a final random sample of farm households was drawn. Households qualified to be included in the sample if they were located in the rural areas within the study sites and were involved in maize production (among other crops) for at least two years preceding the survey year.

Household interviews were conducted using a semi-structured questionnaire eliciting information on household socioeconomic and demographic aspects, crop and livestock production and marketing information, crop and livestock production inputs, historical land management practices, soil and water conservation practices, and other relevant issues such as access to information, infrastructure, group membership and participation in land markets. As already mentioned population density data were obtained from the KNBS 2009 Population and Household Census (Republic of Kenya, 2010). Further, soil samples were collected from the largest maize field in every sampled farm household, following a standardized soil sampling protocol. Organic carbon was determined according to the Nelson and Sommers method (Nelson and Sommers, 1986). Total nitrogen was determined following the procedure in Bremner and Mulvaney (1982). The Melich III extraction method was used to determine exchangeable bases, micronutrients and available P. This is because it allowed analysis of multiple elements from the extractant using inductively-couple plasma spectroscopy (ICP) (Mehlich et al., 1984). Soil pH and EC determination was done in 1:1 water suspension. Reactive carbon was determined using the calorimetric method following Weil et al. (2003). To determine soil texture, samples were first pre-treated to remove organic matter, salts and any other cementing elements present (Bowman and Hutka, 2002) followed by fractionation, drying and weighing of the sand, silt and clay.

## 3 Conceptual framework and estimation procedures

Farmers strive to maximize utility derived from farm and non-farm income generation activities. It is assumed that farmers would allocate scarce resources including available family labour among these activities depending on the expected income from each of these options. Just like any other farmers, farmers in densely populated areas face constraints associated with the quality and quantity of the conventional inputs: land, labour and capital.

Farmers’ behavior may be affected by population density in indirect ways that are not fully captured by variables typically found in a farm production function or available in single-year surveys. For example, population-related land scarcity may influence prior years’ cultivation practices (e.g., whether to fallow the field, how long to have fallowed the field in the past, whether to use crop rotations on the plot), which may affect current period crop yields. It is also possible that population density may be inversely correlated with the availability of family or community land on which to expand in the future, which may influence farmers to intensify their scarce land in more sustainable ways, at least along some range of the population density distribution. The effects of population density on behavior may also differ at different ranges of the population density distribution, warranting tests of non-linear relationships. Crop yields are therefore conceptualized to be influenced by conventional inputs, soil quality and other exogenous drivers such as population density, market access, institutional factors, and farmer socioeconomic attributes.

Soil quality is a broad and complex phenomenon that incorporates soil physical, chemical and biological characteristics and available nutrients. The interactions between the four aspects of soils are important in determining the overall health of agricultural soils. Soil biological status is indicated by the level of soil organic matter (SOM) which is usually an indicator of overall soil quality (Horneck et al., 2011). SOM usually stores plant micro and macro nutrients and makes them readily available to crops (Musinguzi et al., 2013; Bationo et al., 2007). SOM also enhances yield response to fertilizers. SOM is so critical such that when farmers apply fertilizers to soils with extremely low levels of organic matter, most of the applied nutrients leach away making them unavailable to crops and making fertilizer application less economically viable (Marenya and Barrett, 2009; Musinguzi et al., 2013). The level of SOM is commonly measured through the level of Total Organic Carbon in soils. Also, the level of Reactive Carbon (RC), which is the amount of carbon that is easily oxidable is increasingly being used as an indicator of Organic Carbon. The soil chemical attributes can be best captured through the Cation Exchange Capacity (CEC) and pH. CEC is a measure of the soils’ ability to retain and supply positively charged nutrients (e.g. Calcium, Magnesium, Pottasium and Ammonium) (Horneck et al., 2011). Soil pH indicates the concentration of Hydrogen ions and consequently the soil acidity, an important determinant of nutrient availability, biological processes and optimum plant growth. Soil physical attributes are normally indicated by the texture of the soils which again determine the capacity of soils to hold water and nutrient and support biological processes. The interaction between soil biological, chemical and physical characteristics will eventually determine the quantity of nutrients held in the soil and the availability of these nutrients to plants. This in return determines plant growth and eventually crop yields.

The soil quality parameters discussed above can be influenced by both endogenous and exogenous factors. Endogenous factors relate to soil properties that are responsive to farmers’ behavior such as soil management practices (use of crop residue, agroforestry, use of inorganic fertilizers, and fallowing). Exogenous factors include climatic conditions, population density and institutional factors. Crop yields are influenced by the levels of conventional inputs such as land, seed, fertilizer and water, as well as labor and capital inputs, whose availability may change as population density increases.

Analytically, crop output can be modeled based on the neo-classical production function (Douglas, 1976). The most important staple food crop in the study area is maize and therefore the study focuses on this crop. Maize yields in farm *i* can be expressed as:

(1) Y=f(L,M, S, F)

where Y is the maize equivalent yield in Kg/Ha in the main maize plot of household *i* which is computed as the total maize output divided by the size of the plot, while accounting for inter-crops grown on the plot using the relative price ratio with maize following the Liu and Myers (2009) approach which converts the yield of other crops intercropped with maize to maize equivalent^2^; *L* is the size of maize plot (Ha), *M* is the Labour input (MD/ha), S captures the quality of seeds (Hybrid or otherwise) and *F* is the quantity of fertilizers applied.

When considering only conventional inputs in a standard translog production function and the direct effect of population density on maize yields, the linearized production function takes the form:

(2) $$\text{l}\text{n}(Y)=\;\propto +\sum_{i}\beta_{i}ln\left(Z_{i}\right)+\sum_{i}\sum_{j}\text{ln}\left(Z_{i}\right)\text{ln}\left(Z_{j}\right)+\sigma PD+\gamma {PD}^{2}+\varepsilon$$

$Z_{i}$ is a vector of the standard production inputs: size of the plot under maize (L), labour input (M), quantity of seeds (S), and quantity of fertilizer (F). The labour input is computed as the sum of family and hired labour used in the maize production process. The quantity of fertilizer applied to each plot was separated into the Nitrogen and Phosphorous components following Sheahan et al. (2013), considering that these are the most limiting nutrients in most Kenyan soils. PD captures the village-level population density, whereby the coefficients of population density and its squared term (and γ) will be used to assess the direct effect of population density on maize yields. The squared term helps to determine whether there is a non-linear relationship between population density and maize yields. The unobservable factors that influence Y are captured by ε.

The empirical model presented in (2) could potentially be extended to include the influence of other factors besides the standard production inputs. Soil quality variables were included among the growth inputs because soil attributes have direct effects on agronomic aspects of crop growth and failure to include soil quality in the crop yield response function may therefore bias the estimates in (2) (Ekbom et al., 2013; Gray, 2011). Further, crop yields can also be potentially influenced by household socioeconomic factors, market access factors and other community level factors. Modification of Eq. (2) yields:

(3) $$\text{l}\text{n}(Y)=\;\propto +\sum_{i}\beta_{i}ln\left(Z_{i}\right)+\sum_{i}\sum_{j}\text{ln}\left(Z_{i}\right)\text{ln}\left(Z_{j}\right)+\sigma PD+\gamma {PD}^{2}+\sum_{k}\rho_{k}Q_{k}+\sum_{j}\delta_{j}X_{j}+\varepsilon$$

where $Q_{k}$ is a vector of soil biological, chemical and physical attributes and the available nutrients while $X_{j}$ is a vector of other explanatory variables that may influence maize yields such as household socioeconomic attributes, wage rates, and institutional factors. Eq. (3) also allows for interactions between explanatory variables. Soil quality is potentially endogenous because it depends on factors that cannot be directly accounted for in the model such as weather, geological conditions and farmer skills and therefore may be correlated with the error term. To deal with endogeneity problems a two-step approach was used in estimating Eq. (3). First, each soil quality parameter was estimated as a function of explanatory variables and then the predicted values from the models used as explanatory variables in the production function. Soil quality was modeled in the following generalized form:

(4) $$\;Q_{k}=f(m^{'},\mu ,\;\varepsilon )$$

where $\;Q_{k}(k=1,2,3,4)$ represents the soil quality parameters: Organic carbon (OC), Reactive Carbon (RC), Soil pH (pH) and Plant available Phosphorus (P). $m^{'}$ is a Kx1 vector of regressors, μ is a Kx1 vector of coefficients to be estimated and ε is an error term. The regressors included in $m^{'}$ are the factors that are hypothesized to influence soil quality such as other soil chemical, physical and biological attributes, plot characteristics, management practices, farmer attributes, factors that control for regional fixed effects and population density. For each dependent variable, Eq. (4) was estimated first with only plot-level explanatory variables and then it was expanded to include the community-level explanatory variables. The predicted values used in the production function were those from the expanded model.

The most common methods of estimating yield response to input use that we find in literature are the linearized forms of the translog and Cobb-Douglas production functions. However, these functional forms assume symmetry in the way inputs influence outputs and may not allow for accommodating regressors that may indirectly affect the relationship between inputs and output. To address this shortcoming, Guan et al. (2006) proposed a now widely utilized framework that accounts for the asymmetric influence of different types of inputs on output. This framework categorizes inputs into either *growth* inputs or *facilitative* inputs. Growth inputs, such as fertilizers, land, seeds, and water, affect agronomic and growth aspects of crops directly – the biological aspect of the production function. Facilitative inputs, such as labor and pesticides, play a “facilitative’’ role that influence the relationship between growth inputs and output. In the Guan et al. (2006) framework, the general crop production model presented in (3) may be split into two components in the simplified form:

(5) $$y=H\left(z\right)∙F(x)$$

where **y** is the output, ***z*** represents growth inputs, and ***x*** represents facilitating inputs. The $H\left(∙\right)$ component in (5) represents the attainable yields as determined by the conventional growth inputs: land, seeds, fertilizer and soil quality. The scaling function F(∙) represents factors that indirectly affect yields through their influence on the efficacy of the growth inputs. The facilitative inputs may include household socio-economic characteristics and community level factors such as elevation and population density. Eq. (4) was estimated using OLS regression and the predicted values from this model was used as explanatory variables in Eq. (5) which was estimated using a non-linear regression approach using *nlsur* routine in STATA v13.

We first estimated the reduced form of Eq. (5) which only included standard production inputs and population density (Eq. (2)) to assess the direct effect of population density of maize yields. Second, Eq. (2) was expanded to include the soil quality parameters, using the observed soil quality parameters (treating soil quality as fully exogenous) and then using the predicted values (treating soil quality as endogenous). These alternative models enable us to assess the potential bias associated with endogeinity of soil quality. Finally, the full model was estimated to account for potential interactions between inputs, soil quality, and population density. The output elasticities evaluated at the mean values were also estimated to identify the marginal productivity of inputs. The performance of the models were assessed using Likelihood Ratio tests.

## 4 Results

### 4.1 Descriptive analytical results

Table 1 presents mean values of key variables used in the study, estimated for each population density quartile (Columns 1–4) and then for the entire sample (Column 5). These results were generated to assess how farm characteristics change with population density growth. Trends in the production variables reveal evidence of Boserupian type of intensification. While farmland is declining with the growth in population density, we observe that all the major inputs: fertilizer, labor, manure and capital, are increasing with population density. Results indicate high levels of adoption of terracing practice, use of hybrid varieties and production of cash crops, decline in the use of crop residues, fallowing, and agricultural mechanization as population density rises. Declining farm sizes is a clear indication of land scarcity driven by population density as people subdivide the scarce arable land to cater for the increasing demand for cropland. In Machakos, available data indicates that farm sizes have fallen by over 50 percent between 1978 (Mortimore and Tiffen, 1994) and 2015.

**Table 1:** Description of Variables used in the Estimations.

Variable	Description of the variables	Population Density Quartiles				
		1[Lowest]	2	3	4[Highest]	Overall
*Maize Production variables*						
MZYLD	Maize yield (Tons/ha)	1.2	1.1	0.9	1. 1	1.1
LAND	Amount of land owned (Ha)	0.6	0.9	0.6	0.4	0.6
PLOTSIZE	Plot area under maize (Ha)	0.4	0.3	0.3	0.3	0.3
N	Quantity of Nitrogen applied (Kg/ha)	53.2	72.9	45.7	58.7	57.4
P	Quantity of Phosphorus applied (Kg/ha)	34.7	51.0	26.7	33.0	36.2
MAN	Quantity of manure applied (Tons/ha)	3.3	1.9	2.1	1.8	2.2
TOTLAB	Labour input per (Mandays/Ha)	139.3	122.9	140.9	153.5	139.3
HIRELAB	Hired labour input (Mandays/Ha)	108.2	96.5	77.7	74.2	89.3
FAMLAB	Family labour (Mandays/Ha)	102.4	71.6	77.4	89.3	85.1

*Management Practices*						
TERR	Terracing implemented (Yes = 1)	0.9	0.7	0.6	0.5	0.7
RESID	Incorporation of crop residues(Yes = 1)	0.5	0.4	0.5	0.5	0.5
CASHCROP	Farmer growing cash crops (Yes = 1)	0.8	0.7	0.5	0.7	0.7
HYBRID	Farmer planted hybrid variety (Yes = 1)	0.9	0.8	0.8	0.8	0.8
MECH	Mechanized land preparation (Yes = 1)	0.04	0.07	0.01	0	0.03
SBURN	Slush and burn practice (Yes = 1)	0.1	0.1	0.1	0.21	0.13
FALLOW	Fallow Land in the last 5 years (%)	0.2	0.7	2.0	0.6	0.9
DUR	Duration of plot use (years)	40.1	33.1	32.0	26.2	32.9

*Soil Quality attributes*^a^						
TOC	Total organic Carbon (% value)	1.7	1.5	2.0	2.11	1.8
PAP	Plant available Phosphorus (ppm)	12.1	17.4	21.1	13.0	15.9
EC	Electrical Conductivity (ms/Cm)	0.07	0.06	0.08	0.09	0.07
SAND	Sand content in soil (%)	55.6	62.0	59.9	51.0	57.2
pH	Soil pH-H_2_0-1:2.5	5.8	5.9	5.8	5.5	5.7
PLOTDIST	Walking time to the plot (Minutes)	5.3	3.9	6.5	4.0	4.9

*Demographic attributes*						
AGE	Age of household head (Years)	61.2	55.9	53.7	51.2	55.6
HHSIZE	Household size (Adult Equivalents)	2.8	2.9	3.5	2.8	3.0
EDUC	Household head education level (Yrs)	7.9	7.8	8.8	8.4	8.2

*Community level factors*						
POPDENS	Population density (Persons/Km^2^)	507	543	654	843	635
MZTRANS	Cost of transporting maize (Ksh/Km)	53.4	89.1	72.5	38.3	63.5
EXTDIST	Distance to extension service (Km)	5.4	5.1	6.3	5.9	5.7
NCPB^b^	Distance to the nearest NCPB depot (Km)	10.4	9.8	16.8	20.2	14.3
WAGE	Wage rate at village averages (Ksh/Md)	194.5	186.0	148.9	117.6	162.1
DAPRICE^c^	Price of DAP fertilizer (Ksh/Kg)	77.0	79.6	77.9	76.3	77.5
DISTOWN	Distance to the nearest town (Kms)	4.6	4.3	2.9	4.6	4.2
ALT	Altitude (meters above sea level)	1,718	1740	1846	1767	1767
TENURE	Land tenure (Secure = 1)	0.4	0.3	0.2	0.2	0.3

a These values can be compared with the critical values (Aune and Lal, 1997; Weeda, 1987): TOC-2%; N-0.2% PAP-20ppm.

b The National Cereals and Produce Board (NCPB) is a government parastatal that purchases grains and also supplies fertilizer.

c DAP refers to Diammonium phosphate fertilizer.

**Fig. 2:**
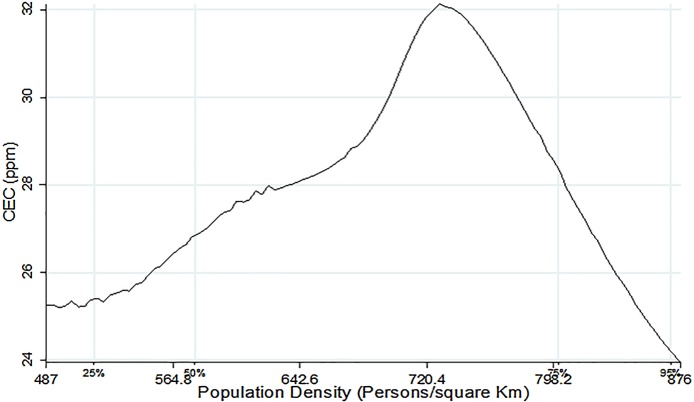
Population density vs Cation Exchange Capacity.

**Fig. 3:**
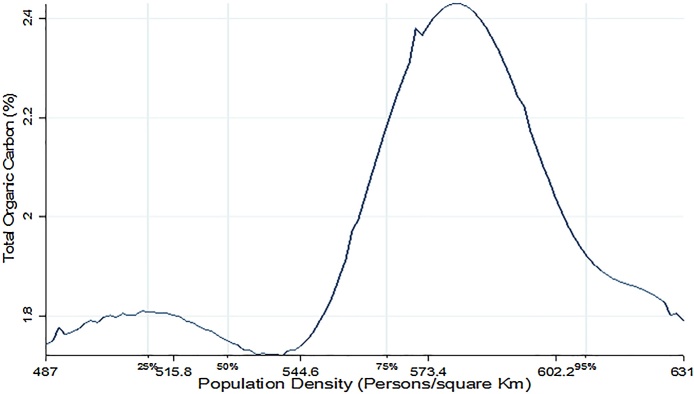
Population density vs Organic Carbon.

**Fig. 4:**
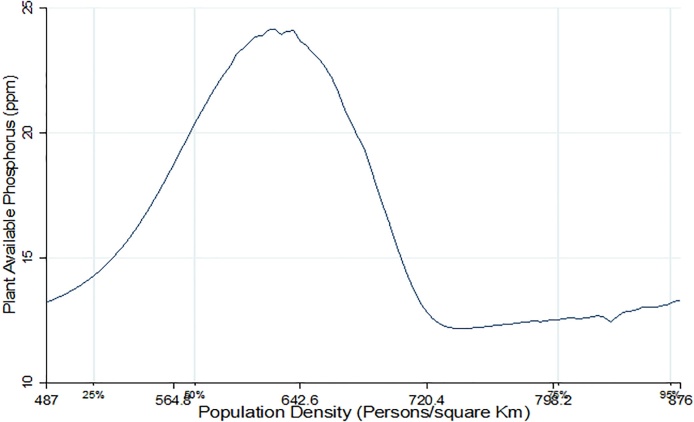
Population density vs Plant Available Phosphorous.

Fig. 2, Fig. 3, Fig. 4 present non-parametric bivariate regression curves relating population density on the x-axis with soil quality parameters on the y-axis. This method is advantageous in that it does not impose functional form in the bivariate relationship between variables, however, it does not allow inclusion of other controls that influence farm productivity. Consequently, the non-parametric results should only be interpreted as a relationship and they do not provide causal effects. As shown in Fig. 2, Fig. 3, Fig. 4 all the three soil quality parameters (OC, CEC and P) first increased with population density growth, reaching a saddle point at approximately 600 persons/km^2^, and then start to decline. At lower population densities soil quality seems to be improving, consistent with the findings of Tiffen et al., (1994). However, beyond the 600 persons per Km^2^ population density threshold, the soil quality starts to decline. The saddle points in Fig. 2, Fig. 3, Fig. 4 coincide with the population density thresholds after which farm productivity starts to decline as reported in earlier studies (see Josephson et al., 2014; Muyanga and Jayne, 2014 and Ricker-Gilbert et al., 2014). This could imply that the decline in farm productivity that has been reported in these studies is caused by deterioration in soil quality as population density rises.

**Fig. 5:**
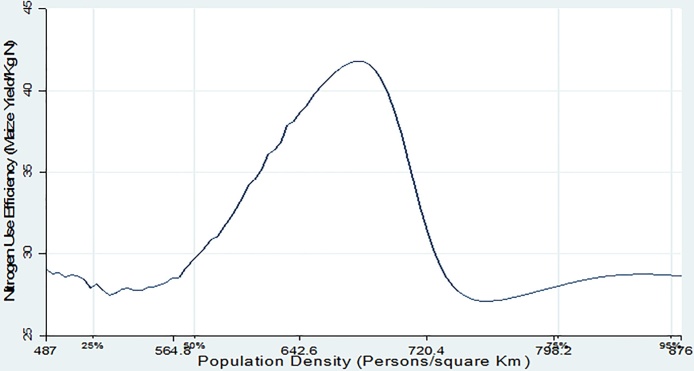
Population density vs Nitrogen use efficiency.

**Fig. 6:**
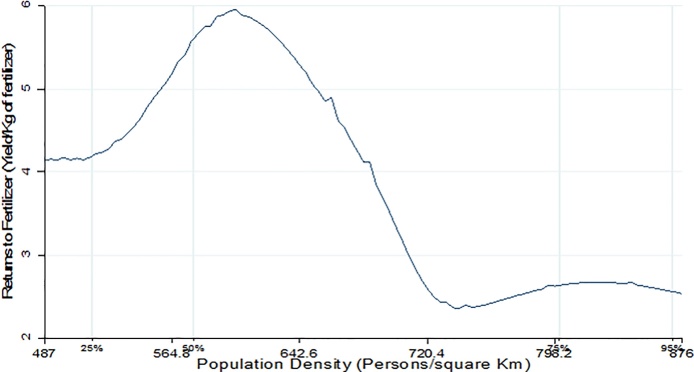
Population density vs Maize Yield.

**Fig. 7:**
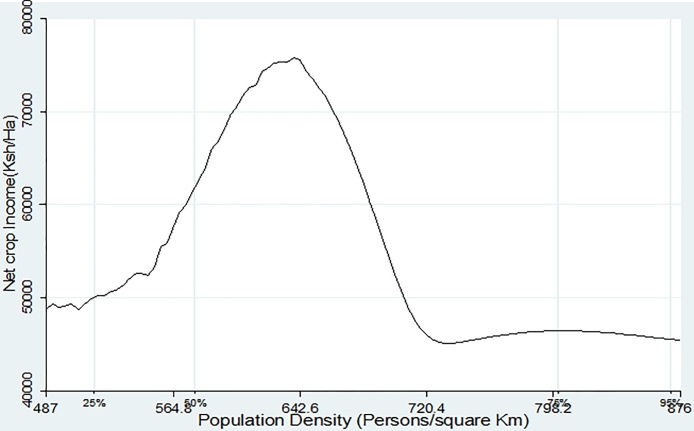
Population density vs Net crop income.

In Fig. 5, Nitrogen Use Efficiency (NUE) first increases with population density then declines. This is consistent with results by Marenya and Barrett (2009) who found a soil fertility threshold below which crop productivity responds weakly to input application. Nitrogen use efficiency relates to the ability of crops to absorb N and utilize it to generate yields, and is measured by the quantity of maize output (Kg) per Kg of Nitrogen applied. NUE is highly influenced by soil parameters such as texture, pH and the level of plant available Phosphorus (P). Fig. 6 shows that returns to fertilizer use (which was measured by the maize yield per kilogram of inorganic fertilizer applied) starts to decline after the 50th population density percentile or at approximately 600 persons per Km^2^. This is the point where soil quality starts to decline. Finally, Fig. 7 depicts a nonlinear relationship between crop income (KSh/ha) and population density with a maximum saddle point at approximately 600 persons/Km^2^. The non-parametric regression results show a clear picture indicating that population density triggers agricultural intensification which at first leads to improvement in soil quality and crop yields. However, as population density reaches a certain threshold, agricultural intensification is no longer sustainable because soil fertility deteriorates, leading to low yield response to fertilizer application and eventually low farm incomes as a result of poor yields.

### 4.2 Econometric estimation results

We now turn to presenting estimation results generated through the two step approach described in the methodology section. The multiple regression results are presented in Table 2 while production function estimation results are presented in Table 3.

**Table 2:** Multiple Regression Coefficients for Determinants of Soil Quality Parameters.

	OC		RC		pH		P	
	MODEL-I	MODEL-II	MODEL-III	MODEL-IV	MODEL-V	MODEL-VI	MODEL-VII	MODEL-VIII
Size of Maize Plot (Ha)	−0.200	−0.12	0.002	0.001	−0.08	−0.02	−0.74	−3.15
Duration Plot used (Yrs)	0.0005	0.001	−1.2E-05	−0.00002	0.005	0.001	−0.01	−0.01
Proportion of land left fallow	0.057^*^	0.05^*^	0.0005	0.0005	0.002	0.02	−0.92	−0.51
Terraces Implemented (Yes=1)	0.065	0.06	−0.001	−0.001	−0.07	−0.02	3.21	3.13^*^
Mechanized Land preparation (Yes=1)	−0.969^***^	−0.68^***^	−0.011^***^	−0.008^*^	0.38^*^	0.26	8.45^*^	−1.29
Use of Crop residues (Yes=1)	0.054	0.01	0.001	−0.0002	−0.10	−0.05	1.19	0.92
Quantity of Manure used (Kg/Ha)	0.045^***^	0.03^*^	0.00015	0.0003	3E-05^***^	0.00002^**^	−0.69	0.72^**^
Quantity of Nitrogen (Kg/Ha)	−0.002^**^	−0.001	7.4E-06	0.00,001	0.003	0.0001	0.04^**^	0.03
Quantity of Phosphorous (Kg/Ha)	0.002	0.001	3.7E-07	−0.000001	0.002	−0.0002	−0.04	−0.03
Clay content in the soil (%)	0.008^**^	0.01^**^	0.0002^*^	0.000055	−0.02^***^	−0.01^***^	−0.05	0.18^**^
Soil pH	−0.082	−0.04	0.005^***^	0.007^***^	–	–	8.60^***^	8.12^***^
Farm has Cashcrop (Yes=1)	−0.188^**^	−0.18^**^	0.015^***^	−0.00005	0.14	0.13	−1.83	2.53
Total Organic Carbon (%)			0.003^***^	0.015^***^				
County Dummy (Machakos=1)		−0.03		−0.009^*^		−0.04		10.81^**^
Population Density (Persons/Km^2^)		0.01^**^		−0.001^***^		0.01^**^		0.94^***^
Population Density Squared (Persons/Km^2^)		−1.2E-05^**^		4.2E-07^***^		−0.00001^**^		−0.0006^***^
Proportion of land under crops		0.36^**^		0.003		−0.08		−1.34
Distance to Cereals Board (Km)		0.01^**^		−0.00009		0.01		−0.16
Cost of Transporting Fertilizer (Ksh/50 Kg bag/km)		0.0004^*^		−0.00001		−0.0001		0.00
Village Wage rate (KSh/Manday)		−0.002		−0.00002		0.004		0.20^***^
Distance to nearest extension services (kms)		−0.01		−0.00016		−0.02^**^		0.00
Price of DAP Fertilizer (KSh/Kg)		0.02		−0.00021^**^		0.01^*^		−0.16
Land Tenure (Secure=1)		0.03		−0.003^*^		0.06		−1.12
Number of Adults in the Household		0.04		0.00004		0.01		−0.98
Education level of the household head (Years)		0.01^*^		0.00005		−0.01^*^		0.24^**^
Age of the Household Head(Years)		−0.002		0.00002		0.003		0.06
Distance to nearest town (Km)		−0.01		0.00,030^*^		−0.02^*^		0.01
INTERCEPT	2.189	−4.24^***^	0.013	0.195^***^	6.28^***^	0.94^***^	−32.19^***^	−398.48^***^
R^2^	0.13	0.34	0.41	0.48	0.08	0.20	0.18	0.44
F	3.42	5.09	14.78	9.22	1.70	2.28	5.05	8.13
Number of Observations (N)	290	290	290	290	290	290	290	290

*,**,*** parameters are significant at the 0.1, 0.05 and 0.01 levels respective. OC = Organic Carbon, RC = Reactive Carbon P = Plant available Phosphorous.

**Table 3:** Maize Yield Response Estimation results.

	Standard Model (SM)		Exogenous Model (EM)		Endogenous Model (ENM)		Full Model (FM)	
	Coef.	Std. Err.	Coef.	Std. Err.	Coef.	Std. Err.	Coef.	Std. Err.
Ln_plot size (Ha)	−2.32^***^	0.338	−0.07	0.0592	−0.19^**^	0.098	−0.12^*^	0.077
Ln_plot size squared	−1.03^***^	0.219	−0.06	0.0373	−0.11	0.062	−0.06^*^	0.040
Ln_Nitrogen input (N)	2.22^***^	0.316	0.03	0.0554	0.17^**^	0.090	0.15	0.094
Ln _Nitrogen Input squared	−0.30^***^	0.053	0.00	0.0090	−0.01	0.015	0.002	0.010
Hybrid seeds (Yes = 1)	1.20^***^	0.287	0.05	0.0481	0.04	0.080	−0.08^***^	0.050
Ln_Phosporous input	−0.26	0.303	0.09	0.0500	0.06	0.080	0.01	0.108
Ln_Phosporous input sq.	0.03	0.059	−0.01	0.0096	−0.02	0.016	0.00003	0.012
Ln_Manure	0.73^***^	0.129	0.05	0.0220	0.01	0.036	0.03	0.023
Ln_Manure squared	−0.06^***^	0.015	0.001^*^	0.0026	0.00	0.004	−0.001	0.003
Pop. density (Psn/Km^2^)	1.15	1.581	−2.65^***^	0.644	−1.83^***^	0.903	−1.81^***^	0.773
Pop. Density squared	−0.09	0.122	0.21	0.050	0.14	0.069	0.14^***^	0.059
Ln_Labour	0.01	0.007	0.001	0.003	0.004	0.004	−0.0003	0.003
Ln_Labour squared	−0.004^*^	0.002	−0.001	0.001	−0.002	0.001	−0.0004	0.001
Ln_Organic Carbon(OC)			0.74	0.0594	−0.21	0.205	1.93^***^	0.601
Ln_Reactive Carbon(RC)			−1.33	0.0645	−0.77	0.199	−0.64^***^	0.243
Ln_Soil pH			1.92	0.1211	1.80	0.201	2.69^***^	0.129
Ln_Plant Available P			−0.04	0.0309	−0.13	0.051	0.15	0.135
Ln_Land * ln N input							−0.003	0.035
Ln_Land * P. input							0.01	0.038
Ln_Land * ln Manure							0.01	0.007
Ln Pop.Density^*^ Ln_OC							−0.001	0.001
Ln Pop.Density^*^ Ln_RC							−0.001^***^	0.0004
Ln Pop.Density^*^ Ln_PAP							−0.001^***^	0.0002
Ln_Nitrogen^*^Ln_OC							−0.21^**^	0.102
Ln_Phosphorous^*^Ln_OC							−0.01	0.110
Ln_No. of years of fert. use							0.0002	0.001
Ln_Land holdings							−0.01^***^	0.004
Ln_Wage rate							−0.005	0.012
Constant	−4.79	5.125	7.53^***^	2.089	4.82^***^	2.932	4.75^*^	2.540
Number of Observations	290		290		290		290	
R-Squared	0.68		0.74		0.84		0.89	

* Parameters are significant at the 0.1 level; *; ** parameters are significant at the 0.05 level; ***parameters are significant at the 0.01 level.

In the soil quality regression models, for every dependent variable we present results from two models generated from Eq. (4). In each case, we first present results from a model that included only plot level explanatory variables and then results from a model that incorporates exogenous regressors that control for fixed effects. Across the four dependent variables, the coefficient of determination (R^2^) presented in the second last row of Table 2 indicate that controlling for fixed effects improves the explanatory power of the models. Total organic carbon and Plant available Phosphorous were the soil parameters that are greatly influenced by community level factors with 0.24 and 0.26 percent improvements in R^2^ respectively. Reactive carbon and soil pH on the other hand were least influenced by community level factors but more by inherent plot level attributes. In all the models, we rejected the null hypothesis that all the coefficients were jointly equal to zero as shown by the significant F values in the last row of Table 2.

The results presented in column 2 of Table 2 demonstrate that Organic carbon is positively influenced by fallowing, clay content and quantity of manure but negatively by agricultural mechanization and the presence of cash crops on the farm. Fallowing facilitates the build-up of soil organic matter because the practice allows soils to recover after a cropping season. However, as a result of population induced land scarcity, fallow periods shorten and eventually disappear as they give way to yearlong cropping cycles (Boserup, 1965). Mechanized land preparation had a negative influence on TOC possibly because it disturbs the soil structure hence accelerating the breakdown of organic carbon. Consistent with findings by Jindaluang et al. (2013), the clay content had a positive effect on TOC. Soils which are rich in clay normally preserve organic matter by slowing down the decomposition process. As indicated in Table 1, soils in the research area are mainly Sandy and therefore this could compromise the capacity of the soils to preserve organic matter. Farms where cash crops (tea and coffee) were grown also had lower TOC. This finding reflects farmer priorities in the decisions on how organic manure is allocated across plots within the farm. Where a farmer has both food and cash crops, they would allocate more organic fertilizers to the plots with cash crop and less to the plots with food crops.

Column 3 of Table 2 shows that beside the plot level drivers, organic carbon is also significantly influenced by exogenous community and regional level determinants. Consistent with the non-parametric regression results (Fig. 3) population density and its squared term had a positive and negative influence on organic carbon respectively, implying that at lower population density organic carbon increases with population density but then declines at higher densities. Population density therefore has a non-linear relationship with soil organic carbon in this area of Kenya, which give us some clues about the driving factors behind the non-linear relationship between crop output per hectare and population density as observed in other studies (Ricker-Gilbert et al., 2014; Muyanga and Jayne, 2014; Desiere and D'Haese, 2015).

The coefficient of the proportion of land under crops was positive and significant. Increasing the distance from a National Cereals and Produce Board (NCPB) by one Kilometer was associated with an increase in organic carbon content by 0.014 percent. The NCPB is a government-operated institution responsible for buying cereals as well as distributing inputs, and its positive coefficient may signify that NCPB locates its depots in primarily areas of favorable soil conditions. The maize transport cost variable was found to be associated with higher levels of soil organic carbon, signifying that more remote areas may have experienced less land pressure and soil degradation than accessible areas. The price of DAP, a common fertilizer used in the research area was found to positively influence organic carbon. High prices of inorganic fertilizers could also imply low access to fertilizers which discourages the use of inorganic fertilizers and encourage the use of manure.

Column 4 and 5 of Table 2 presents results on the determinants of reactive carbon (RC), which is the fraction of total organic carbon that is readily oxidable through the mineralization process. RC has a short turnover time and is more sensitive to changes in land management practices (USDA, 2014). Soil pH was positively related to the level of reactive carbon. Lower soil pH reduces soil microbial activity hence slowing down the mineralization process, making a larger proportion of carbon to remain in the aggregates and therefore not readily oxidizable. Compared to Kisii, soils in Machakos had lower levels of reactive carbon implying that farming practices in Kisii were accelerating the process of organic carbon break down. This could be explained by the fact that population densities in Kisii are higher that those in Machakos and therefore agricultural intensification was higher in Kisii. Reactive carbon first decreased with population density and then increased, contrally to the trends in total organic carbon. The decline in reactive carbon as population density grows is a signal for deteriorating soil quality attributed to soil organic matter which may limit soil water holding capacity, microbial activity and nutrient availability.

The determinants of soil pH are presented in column 6 and 7 of Table 2. Results for the model which had plot level explanatory variables only show that clay mineral content had a negative influence on soil pH while the cash crop dummy had a positive effect. Population density had a positive and significant influence on pH which was nonlinear as indicated by the negative coefficient of the squared term of population density. High population density growth is found to be associated with acidification of soils. Lack of extension services as indicated by the distance to the extension service providers was found to have a negative effects on soil pH. Farmers who lack extension advice are more likely to have acidic soils. Where extension advice is lacking, farmers tend to apply almost the same types of acidifying fertilizers every season. Given that farmers in the study area operate within an environment of limited extension advice and limited access to soil testing services, they default into blanket fertilizer applications ignoring heterogeneity of farm plots and spatial differences in soil fertility and nutrient requirements.

The results of model VII in Table 2 show the determinants of plant available phosphorous (P), which was positively influenced by the amount of manure applied. Manure is rich in phosphorus and unlike inorganic fertilizer it does not lower soil pH and therefore prevents Phosphorous fixation. As expected, soil pH had a strong positive effect on P. At lower pH levels, phosphorus is normally bound through the formation of insoluble compounds (Truog, 1930). When we control for fixed effects (model VIII), soil conservation through terracing had a positive effect on P. Soil conservation preserves the top soil and therefore helps in the buildup of soil nutrients and improvement in crop yields (Ekbom et al., 2013; Willy et al., 2014). Population density had significant direct and indirect effects on Phosphorous. The coefficients of population density and its squared term had positive and negative effect on P respectively, implying improvement in soil P at lower population densities which deteriorates after the density reaches certain a turning point. The indirect effect is seen through the positive effect of wage rate on plant available phosphorous. An increase in the price of DAP was associated with a decline in the amount of P.

Table 3 presents maize yield response results obtained from estimating the asymmetric production function (Eq. (5)). Table 3 present results of the four models we estimated, as explained in the methodology section. Based on the Likelihood Ratio test results (Table 5), we find an improvement of the restricted model by adding soil quality parameters and that the full model performed best. Column 2 of Table 3 presents coefficients of the restricted model, which only included the standard production inputs, population density and its squared term. The coefficients of population density and its squared term are not significant implying that population density does not have a direct effect on maize yields. Column 3 presents results of a model that includes soil quality as covariates to assess the indirect effect of population density. This is an endogenous model since we used the observed soil quality parameters rather than the predicted parameters. The next model controlled for endogeinity by including the predicted soil quality parameters. In this model, the coefficients of organic carbon, soil pH are positive implying that improvement in soil quality is associated with maize yield increment and that raising soil pH is also associated with improvements in crop yields. Comparing the coefficients with those from the endogenous model, it is clear that failing to control for endogeinity would have biased the coefficients of plot size, seed type, Nitrogen and Labour.

In the last column of Table 3, we present results of a model that included all the variables in the first three models and also interaction terms and the predicted values of other endogenous drivers of maize productivity such as wage rates and land holdings, which are closely correlated to population density. For a more intuitive interpretation of the production function results, we computed the mean elasticities of selected covariates presented in Table 4. The coefficients of plot size and its squared term were jointly significant at 0.05 level and the output elasticity of land was −2.30. This indicates an inverse land size-yield relationship a finding that concurs with those of (Ünal, 2008) and Pieralli, (2014). Some of the popular arguments to explain the inverse land size-yield relationship found in literature include crop diversity (Griffin et al., 2002); land and labour market imperfections (Ali and Deininger, 2014; Holden and Fisher, 2013), and high labour input (Ünal, 2008). Better soil quality may make the inverse land yield effect even more prominent as seen in the highly statistically significant coefficients of the interaction terms between plot size and all the soil quality parameters. The output elasticity of labour was 3.08. The low labour productivity implies labour abundance that is characteristic of densely populated areas. As population density increases, labour productivity decline as a result of substitutability between labour and capital (Binswanger and Pingali, 1998).

**Table 4:** Elasticities of Selected Variables.

	Standard Model (SM)		Exogenous Model (EM)		Endogenous Model (ENM)		Full Model (FM)	
Variable	Elasticity	SE	Elasticity	SE	Elasticity	SE	Elasticity	SE
Land	−1.75	0.24	−2.35	0.042	−2.27	0.069	−2.30	0.139
Labour	3.09	0.002	3.08	0.001	3.09	0.001	3.08	0.001
Nitrogen	2.99	0.095	2.33	0.017	2.34	0.026	2.35	0,071
TOC			0.90	0.109	0.75	0.182	1.32	0.351
P			1.47	0.013	1.46	0.012	1.52	0.052
pH			1.73	0.07	2.08	0.06	2.01	0,006

SE = Standard error.

**Table 5:** Results on Likelihood Ratio test.

Comparison	Models	LR χ^2^	AIC Estimate
(1)	Reduced form model	1010.2***	1372.3
	Endogenous model		856.9
(2)	Exogenous model	645.5***	920.1
	Full model		762.2
(3)	Reduced form model	723.7***	1372.3
	Exogenous model		920.1

Nitrogen exhibited increasing returns as shown by the Elasticity of 2.35, implying that increasing the amount of N by 10% would increase maize yields by 23.5% indicating maize production exhibits increasing returns to N. Nitrogen has been found to be one of the most limiting macro-nutrients in most Kenyan soils (Ekbom et al., 2013; Marenya and Barrett, 2009), a fact this result confirms. The elasticity of Phosphorous was positive and significant. Increasing the quantity of P input by 10% results into an increase in maize yields by 15.2%. Soils in Machakos have been particularly found to be naturally deficient in P and therefore addition of P will lead to increment in maize yields.

Soil quality was found to have substantial influence on maize output. Among the soil quality parameters, soil pH had the highest elasticity (2.01) followed by Plant available Phosphorous (1.52) and eventually total organic carbon (1.32). Improving soil pH would improve soil biological processes as well as availability of critical nutrients, which would be otherwise bound under low pH conditions. Plant available Phosphorous is the component of P that can be assimilated to plants and therefore would boost yields as well. Finally, improvement in soil organic carbon would ensure a rich pool of nutrients and their availability to crops would eventually boost yields. Generally, improved soil quality improves the yields by enhancing the ability of the crops to absorb nutrients more efficiently hence boosting the crop yield response to fertilizers (Ekbom et al., 2013; Marenya and Barrett, 2009; Xu et al., 2009).

Also of interest was the interaction term between population density and soil quality. Theoretically, it is expected that as population density increases, there is deterioration in soil organic carbon. We find the coefficients of the interaction term between Organic carbon and population density positive and significant. This implies that if soil fertility would increase as population density increases, the result would be improvement in maize yields. The interaction term between Nitrogen input and Organic Carbon was also positive and significant. This implies that adding Nitrogen to soils with high organic carbon content would lead to improved crop yields. This highlights the importance of sustainable agricultural intensification in the face of growing population density.

## 5 Conclusions and policy implications

The current study finds that although sustainable agricultural intensification can be possible at relatively low population densities, the gains associated with the Boserupian kind of intensification start to diminish as population densities go beyond the 600 persons/Km^2^ threshold. Beyond this population density threshold, sustainable agricultural intensification declines with consequences on agricultural productivity. Endogenous technological change seems sufficient to drive agricultural growth at low population density, as also observed by Binswanger and Pingali, (1998), but may be limited at higher densities.

Our results reveal that at population densities beyond 600 persons/Km^2^, farmers engage in excessive agricultural intensification where fallows almost disappear. In addition there is continuous use of the same inorganic fertilizers without substantial efforts to replenish soil organic matter. As a result, the soil quality substantially declines as indicated by high levels of soil acidity, deterioration of soil texture (soils become sandier), and decline in inherent soil fertility (as measured by Total organic Carbon and plant available Phosphorus). The decline in soil quality can affect crop yields through a chain of processes. Soil acidity causes critical nutrients to be bound in the soil in forms that are not accessible to the plants as manifested in the low levels of plant available phosphorus. High levels of acidity may also facilitate the increase in the level of toxic elements such as aluminium and manganese. A combination of soil acidity, poor soil texture, and low plant available phosphorus causes a reduction in the Nitrogen use efficiency and therefore leading to low yield responses to fertilizer use. The low yield response to fertilizer affects crop yields and eventually causes returns to investment in agriculture to decline. Low crop yields in small holder agriculture in Africa are counterproductive particularly to food self-sufficiency goals. Low returns to agricultural land and labour are also likely to discourage investment in agriculture, encouraging exits from farming. In the densely populated areas of Kenya, exiting from agriculture is not a viable option because there are limited opportunities in the non-farm sector which does not grow at a reasonable rate to accommodate substantial numbers of labourers seeking an alternative to the farming sector. This challenge is likely to persist especially because the population in SSA is projected to reach 2.4 billion by 2050 (Haub and Kaneda, 2013), with a projected 330 million young Africans joining the job market in the next two decades (Jayne and Traub, 2016).

Agricultural policies in the future must therefore deal with the issue of unsustainable intensification in densely populated areas for as long as masses in these areas are still trapped in agriculture. There is need for a more focused and careful approach to soil quality management while enhancing strategic institutional support. For instance, acidity was found to be a major problem among the soils in the densely populated areas, a problem that needs to be addressed. One of the major causes of soil acidity was identified as the excessive use of acidic fertilizers such as DAP. Consequently, on-farm soil testing and advisory services on appropriate fertilizer types are encouraged. Measures to enhance access to low cost soil testing services such as subsidized and decentralized soil testing facilities can help to solve the challenge. Although the private sector plays a critical role in providing advisory services, it can be inefficient. Concentrating advisory information mostly on private input dealers may bias such information towards promotion of certain fertilizer types without consideration of specific field conditions. Thus, technical advice on appropriate fertilizer combinations that are able to achieve nutrient replenishment without further deteriorating the soil quality are needed. Awareness on the importance of soil testing and improved access to soil testing facilities by resource-poor farmers is also encouraged. Additionally, incentives to encourage liming of soils, such as improved access to subsidized agricultural lime, can be a critical step. The challenge of organic matter decay can be addressed through encouraging practices that build organic matter such as incorporation of crop residues, avoiding slash and burn practices, and by incorporation of organic manure on the soils.

It is clear that most smallholder farmers in Sub-Saharan Africa are likely to face further declines in productivity, with serious consequences on food security goals. Towards a more firm policy advice, we recommend the quantification of the rate of soil quality deterioration at different population density levels and management practices.

## Footnotes

1 A Ward is fourth in the hierarchy of administrative units in Kenya.

2 The maize yield equivalent for each household is computed by considering all the intercrops in the maize field using the Liu and Myers (2009) approach, where the maize yield equivalent ${(Y}_{m}$) is computed as: $Y_{m}=\frac{\sum Y_{ik}P_{k}}{P_{m}}$ where $Y_{ik}$ is the output of crop *k* in Kgs in household i; $P_{k}$ is the price of crop *k* and $P_{m}$ is the price of maize. The most common intercrops were beans, green grams, pigeon peas and cowpeas.
